# Changes in lips, cheeks and tongue pressures after upper incisor protrusion in Class II division 2 malocclusion: a prospective study

**DOI:** 10.1186/s40510-017-0182-0

**Published:** 2017-09-25

**Authors:** Irmak Partal, Muge Aksu

**Affiliations:** Department of Orthodontics, Faculty of Dentistry, Hacettepe University, Sihhiye, 06100 Ankara, Turkey

**Keywords:** Class II division 2 malocclusion, Perioral pressure, Incisor position

## Abstract

**Background:**

The etiology of Class II division 2 (CII/2) malocclusion focuses on heredity; however lip, cheek, and tongue pressures that are associated with the environmental effect are considered to have an effect. The aim of this study was to evaluate the relation between perioral pressures and the upper incisor inclination in CII/2 malocclusion.

**Methods:**

Twenty patients (8 females, 12 males; mean age 10.29 ± 0.90 years) with CII/2 malocclusion were included in the study group, and 15 patients (5 females, 10 males; mean age 10.56 ± 1.06 years) with Class I malocclusion were included. The upper incisors were protruded with a utility arch (0.016 × 0.022 in. blue elgiloy wire). Perioral pressure assessment was made with the Iowa Oral Pressure Instrument. Upper lip pressure, lower lip pressure, vertical lip pressure, left-right buccal pressures, swallowing, and maximum tongue pressures were measured. Repeated measure ANOVA was used to test the intragroup differences. Intergroup comparisons were made using two-way repeated measure ANOVA with Bonferroni correction. Relationships between the variables were analyzed using rank correlation (Spearman’s rho). The significance for all statistical tests was predetermined at *p* < 0.05.

**Results:**

A significant change occurred in the upper lip pressure, lower lip pressure, and vertical lip pressure; however, significant difference was not found between the groups. Upper lip pressure increased significantly in both groups. In the CII/2 group, lower lip pressure increased after protrusion and decreased after retention, while vertical lip pressure decreased and then increased significantly. Left buccal pressure changes between the groups were not parallel. Right buccal pressure, swallowing, and maximum tongue pressure changes were not statistically significant. Statistically significant correlation was found between U1-NA (mm) and vertical lip pressure (*r* −0.467).

**Conclusions:**

In the CII/2 group, upper lip pressure increased only in retention. Lower lip pressure increased and vertical lip pressure decreased after protrusion. Nevertheless, these changes did not remain stable after the retention period. The difference between groups was not statistically significant at the end of retention.

## Background

The identification of the etiology is quite important for the success of the orthodontic treatment. In the etiology of Class II division 2 (CII/2) malocclusion, genetics is accepted to be the most important etiologic factor. In studies among twins and triplets, strong evidences were obtained regarding the fact that genetics is the fundamental etiologic factor in the development of CII/2 malocclusion [1–3], though other etiologic factors are lip, cheek, and tongue-related environmental factors. Previous studies have shown that the lips and cheeks, rather than the tongue, are the most important environmental factors of teeth position [4–6]. For stable treatment results, it is necessary to determine the effects of those factors in malocclusion.

Lower lip resting pressure is indicated to be more affective on the position of the upper incisors rather than the upper lip [5, 7]. In some studies, high lip line was shown as the reason behind the retroclined position of the upper incisors [8–11], while in others, the hyperactive lip or mentalis muscle were shown as the reason [12–14]. It has been shown that for individuals with CII/2 malocclusion exposed to significantly higher resting lip pressure than those with Class I malocclusion, the high lower lip line and its pressure were found to be related to the retroclination of the upper incisors in CII/2 malocclusion [10]. Oppositely, in another study, it was mentioned that the pressure from the lips is a result of the incisor position [7].

In the early treatment of CII/2 malocclusion, correcting the molar relationship and inclination of the upper incisors are recommended for the initial phase of treatment. Several appliances such as cervical headgear, removable appliances with anterior bite block, or fixed appliances can be used to correct the malocclusion. The study examining the effects of the cervical headgear on tongue pressure is present in the literature [15]. However, there are no clinical studies examining the effects of fixed-treatment mechanics, mentioned above, on perioral soft tissues yet.

The purposes of our study were to evaluate the changes in perioral pressures after protruding the upper incisors and to investigate if there is any relationship between the upper incisor inclination and perioral pressure changes. The null hypothesis is that protruding the upper incisors does not change the perioral pressures.

## Methods

This prospective study was approved by the Ethics Committee of Hacettepe University with the approval number KA-15027. The individuals and their parents were informed about the treatment process, and all of them signed consent forms voluntarily.

The study group was composed of 20 Caucasian subjects (8 females and 12 males), with the mean age of 10.29 ± 0.90 years, who have CII/2 malocclusion and applied to be treated at the Hacettepe University, Faculty of Dentistry, Department of Orthodontics. The inclusion criteria for the study group were (1) horizontal growth direction, (2) deep bite, (3) retroclined upper incisors, (4) cusp to cusp and/or Class 2 molar tooth relation, (5) not having congenitally missing upper incisors, and (6) not having orthodontic treatment before.

A control group was also composed to distinguish the growth effects on perioral pressures when compared to the study group. Fifteen Caucasian subjects (5 females and 10 males) with the mean age of 10.56 ± 1.06 years who had Class I malocclusion with minimum crowding were included. The inclusion criteria for the control group were (1) upper incisors with normal inclination and (2) not using any fixed or removable orthodontic appliance during the study.

The exclusion criteria for both of the groups were (1) having any systemic disease or craniofacial deformity and (2) having any bad oral habits.

### Perioral pressure evaluation

The evaluation of the perioral pressure of the subjects in the study and control groups was done by using the Iowa Oral Performance Instrument (IOPI). The pressures were recorded before the treatment (T0), at the end of incisor protrusion (T1), and after the retention period (T2). Control group records were taken approximately 6 months later from the T1 records, similar to the study group.

**Fig. 1:**
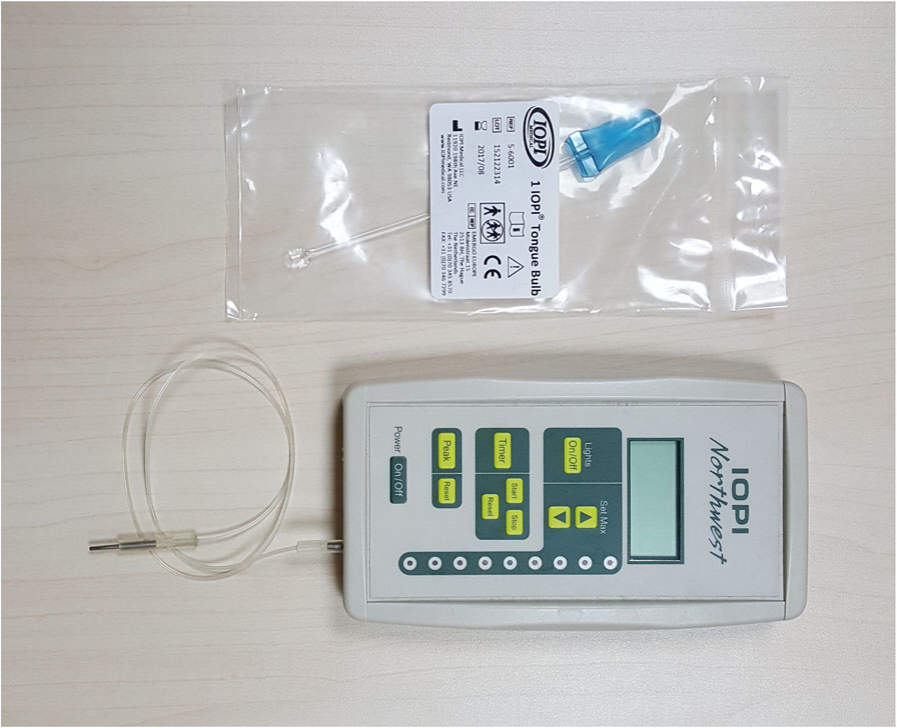
Iowa Oral Pressure Instrument

IOPI is a pressure-sensing circuitry and used in conjunction with a connecting tube and measurement bulb (Fig. 1). The device measures the maximum pressure of the lips, cheeks, and tongue in an air-filled bulb. Each measurement was repeated every 10 s, for three times in total. The pressure values were recorded in terms of kilopascals (kPa), and the average values were calculated according to the arithmetic mean.

During the measurement, comfortable, relaxed, and upright sitting position of the patient was provided to achieve natural head position. All participants were trained by verbal training before taking the perioral pressure records.

The following measurements were done in order to evaluate the perioral pressure:

- Upper lip pressure (ULP): The measurement bulb was located between the upper lip and upper incisors, in the midline between the upper central incisors.
- Lower lip pressure (LLP): The measurement bulb was located between the lower lip and lower incisors, in the midline between the lower central incisors.
- Vertical lip pressure (VLP): The measurement bulb was located between the upper and lower lips, in the midline without contacting the teeth.
- Left buccal pressure (LBP): The measurement bulb was located on the premolar teeth, aligned distal to the left canine tooth.
- Right buccal pressure (RBP): The measurement bulb was located on the premolar teeth, aligned distal to the right canine tooth.
- Swallowing tongue pressure (STP): The measurement bulb was located on the tongue, and the patient was asked to swallow.
- Maximum tongue pressure (MTP): The measurement bulb was located on the tongue, and the patient was asked to squeeze the measurement cap between his tongue and palate.

### Cephalometric evaluation

In order to determine the changes of upper incisors’ inclination (U1-SN angle, U1-NA angle, U1-NA distance), digital lateral cephalometric radiographs were taken in natural head position for the study group at the T0, T1, and T2 stages. The cephalometric measurements were performed by using the 11.8 version of Dolphin software (Dolphin Imaging & Management Solutions, Chatsworth, CA, USA).

### Orthodontic treatment protocol

Orthodontic treatment started with application of a transpalatal arch for increasing the anchorage of the upper molar teeth. Conventional brackets of 0.018-in. slots (Gemini, 3M Unitek, Monrovia, CA) were bonded to the upper incisors. A 0.016-in. and/or 0.016 × 0.016-in. nickel titanium levelling utility arch was applied according to the crowding. After the levelling process, a protrusion utility arch was bent out of a 0.016 × 0.022-in. blue elgiloy wire and was applied. The subjects were observed every 4 weeks. In order to determine if there is enough protrusion, the inclination of the incisors was evaluated clinically. Moreover, whether there was an interference between upper and lower incisors or not was controlled by bringing the mandibula towards to the Class 1 molar-canine tooth relation. When enough protrusion was achieved, a Hawley retainer was applied during the 6-month retention period.

### Statistical analysis

SPSS Statistics software (version 21, IBM Corp, Armonk, NY) was used for the analysis of the data. The normality of the variables is evaluated with the Kolmogorov-Smirnov test. In repetitious measurements, the intra-observer reliability was evaluated with intra-class correlation coefficient. All cephalometric measurements were realized by the first author (….), and the measurements of five patients were repeated within 15 days. In order to evaluate the reliability of the perioral pressures, the measurements that have been repeated before were used.

A power calculation indicated that the achieved power for the study was 0.98. The descriptive statistics were expressed as mean ± standard deviation for continuous variables. The two groups were evaluated using the independent sample *t* test in terms of quantitative variables and chi-square test in terms of categorical variables. In order to observe the difference of the variables within the group according to the time frames, repeated measure ANOVA was utilized. Two-way repeated measure ANOVA was used to examine the main effects of the malocclusion groups within the treatment stages and the interaction effects between them for the pressure measurements. The Bonferroni test was used as post hoc multiple comparisons. Spearman rho correlation coefficient was calculated to find any correlation between the upper incisor position and pressure measurements. The results for *p* < 0.05 were accepted to be significant statistically.

## Results

The data is distributed normally according to the Kolmogorov-Smirnov normality test. While the intra-class correlation coefficient for cephalometric measurements is between 0.972 and 1.000, for the perioral pressure, measurements are between 0.924 and 0.986.

In terms of sex distribution, there was no significant difference between the study and control groups (*p* > 0.05). At the same time, no statistically significant difference between the groups in terms of starting age of treatment could be identified (*p* > 0.05). The duration of orthodontic treatment was calculated as 4 months, retention period as 6 months, and the total duration of treatment as 10 months.

Cephalometric measurements are shown in Table 1. All parameters about upper incisor inclination (U1-SN angle, U1-NA angle, and U1-NA distance) increased significantly (*p* < 0.001, T1-T0, T2-T0, Table 2).

**Table 1:** Cephalometric measurements of the study group

	T0	T1	T2	*p*
	Mean ± SD	Mean ± SD	Mean ± SD	
U1-SN (°)	89.49 ± 5.69	108.91 ± 5.25	106.71 ± 6.00	0.000*
U1-NA (°)	8.69 ± 5.37	28.70 ± 4.67	26.41 ± 3.99	0.000*
U1-NA (mm)	−1.39 ± 1.62	4.90 ± 1.51	4.65 ± 1.24	0.000*

*p < 0.001

**Table 2:** Cephalometric measurement changes of the study group in different treatment stages

	T1-T0		T2-T1		T2-T0	
	Mean difference ± SD	*p*	Mean difference ± SD	*p*	Mean difference ± SD	*p*
U1-SN (°)	19.43 ± 1.47	0.000*	−2.21 ± 0.97	0.107	17.22 ± 1.43	0.000*
U1-NA (°)	20.02 ± 1.45	0.000*	−2.30 ± 0.90	0.058	17.72 ± 1.31	0.000*
U1-NA (mm)	6.29 ± 0.43	0.000*	−0.26 ± 0.28	1.000	6.03 ± 0.30	0.000*

*p < 0.001

There are statistically significant changes observed for the measurements of ULP (*p* < 0.001), LLP (*p* < 0.01), and VLP (*p* < 0.001) (Table 3). The interaction between malocclusion and treatment was seen in LLP, VLP, and LBP (*p* < 0.01) (Table 3).

**Table 3:** Two-way ANOVA results for the pressure measurements

	*p*		
	Malocclusion	Treatment	Malocclusion treatment
Upper lip pressure (kPa)	0.271	0.000**	0.414
Lower lip pressure (kPa)	1.000	0.005*	0.001*
Vertical lip pressure (kPa)	0.149	0.000**	0.002*
Left buccal pressure (kPa)	0.578	0.065	0.002*
Right buccal pressure (kPa)	0.864	0.725	0.224
Swallowing tongue pressure (kPa)	0.751	0.198	0.291
Maximum tongue pressure (kPa)	0.108	0.826	0.503

*p < 0.01, **p < 0.001

The difference between the groups was observed in LBP at the beginning of treatment (*p* < 0.05, T0, Table 4) and in VLP after the protrusion (*p* < 0.05, T1, Table 4).

**Table 4:** Comparison of pressure measurements according to the treatment stages between the study and control groups

	T0			T1			T2		
	Study group Mean ± SD	Control group Mean ± SD	*p*	Study group Mean ± SD	Control group Mean ± SD	*p*	Study group Mean ± SD	Control group Mean ± SD	*p*
Upper lip pressure (kPa)	22.13 ± 3.23	22.17 ± 2.70	0.960	21.68 ± 2.32	23.17 ± 2.13	0.060	24.65 ± 2.90	25.69 ± 3.96	0.377
Lower lip pressure (kPa)	23.15 ± 4.24	23.15 ± 4.61	0.956	26.90 ± 5.95	24.20 ± 4.93	0.163	22.95 ± 4.01	25.73 ± 5.80	0.103
Vertical lip pressure (kPa)	9.38 ± 2.38	8.97 ± 1.92	0.593	7.81 ± 2.52	9.81 ± 1.75	0.013*	9.63 ± 2.79	11.11 ± 2.30	0.105
Left buccal pressure (kPa)	21.93 ± 3.35	18.93 ± 3.21	0.012*	19.00 ± 2.69	20.22 ± 2.88	0.205	20.86 ± 4.07	21.15 ± 3.07	0.820
Right buccal pressure (kPa)	20.90 ± 3.18	20.00 ± 3.26	0.419	19.71 ± 3.34	20.73 ± 3.10	0.364	20.28 ± 4.37	19.66 ± 2.99	0.643
Swallowing tongue pressure (kPa)	23.00 ± 6.71	26.11 ± 12.09	0.339	24.93 ± 7.82	24.75 ± 8.11	0.948	23.00 ± 8.70	22.38 ± 5.06	0.807
Maximum tongue pressure (kPa)	46.48 ± 7.00	49.55 ± 8.75	0.257	44.46 ± 8.49	50.11 ± 8.05	0.055	46.18 ± 9.24	49.44 ± 7.26	0.267

*p < 0.05

The perioral pressure changes in different treatment stages between the study and the control group are shown in Table 5. ULP increased significantly after the retention period in the study group (*p* < 0.01, T2-T1) and also in the control group (*p* < 0.05, T2-T1). These increases for both groups remained stable at the end of the study (*p* < 0.05 for the study group, *p* < 0.01 for the control group, T2-T0). In the study group, LLP increased after protrusion (*p* < 0.01, T1-T0) and then decreased back to the initial values after the retention period (*p* < 0.001, T2-T1). Meanwhile, VLP decreased after protrusion (*p* < 0.01, T1-T0) and then increased back significantly after the retention period (*p* < 0.01, T2-T1). While, in the control group, VLP increased during the study and it was observed that at the end of the study, the value was statistically significant (*p* < 0.01, T2-T0). Moreover, LBP decreased significantly after protrusion in the study group (*p* < 0.01, T1-T0). The significance has not been determined at the RBP, STP, and MTP measurements (*p* > 0.05).

**Table 5:** Statistical analysis of the pressure changes in different treatment stages between the study and control groups

		T1-T0		T2-T1		T2-T0	
		Mean difference ± SD	*p*	Mean difference ± SD	*p*	Mean difference ± SD	*p*
Upper lip pressure (kPa)	Study group	−0.45 ± 0.57	1.000	2.97 ± 0.75	0.001**	2.52 ± 0.83	0.014*
	Control group	1.00 ± 0.66	0.418	2.51 ± 0.87	0.020*	3.51 ± 0.96	0.003**
Lower lip pressure (kPa)	Study group	3.75 ± 1.03	0.003**	−3.95 ± 0.79	0.000***	−0.20 ± 0.98	1.000
	Control group	1.13 ± 1.19	1.000	1.54 ± 0.91	0.308	2.67 ± 1.13	0.072
Vertical lip pressure (kPa)	Study group	−1.57 ± 0.39	0.001**	1.82 ± 0.49	0.002**	0.25 ± 0.47	1.000
	Control group	0.85 ± 0.46	0.216	1.29 ± 0.56	0.084	2.13 ± 0.54	0.001**
Left buccal pressure (kPa)	Study group	−2.93 ± 0.71	0.001**	1.87 ± 0.81	0.084	−1.07 ± 0.79	0.565
	Control group	1.29 ± 0.82	0.375	0.93 ± 0.94	0.980	2.22 ± 0.92	0.062
Right buccal pressure (kPa)	Study group	−1.18 ± 0.63	0.203	0.57 ± 0.80	1.000	−0.62 ± 0.88	1.000
	Control group	0.73 ± 0.72	0.953	−1.07 ± 0.92	0.760	−0.33 ± 1.01	1.000
Swallowing tongue pressure (kPa)	Study group	1.93 ± 1.49	0.609	−1.93 ± 1.72	0.807	0.00 ± 1.82	1.000
	Control group	−1.36 ± 1.72	1.000	−2.38 ± 1.99	0.720	−3.73 ± 2.10	0.255
Maximum tongue pressure (kPa)	Study group	−2.02 ± 1.64	0.678	1.72 ± 1.39	0.678	−0.30 ± 1.73	1.000
	Control group	0.56 ± 1.89	1.000	−0.67 ± 1.61	1.000	−0.11 ± 2.00	1.000

*p < 0.05, **p < 0.01, ***p < 0.001

The relationship between the changes in the inclination of upper incisors (U1-SN angle, U1-NA angle, U1-NA distance) as well as the changes in the ULP, LLP, and the VLP were examined after the protrusion and retention periods. The decrease in the VLP (1.57 kPa) had a negative and moderate correlation (correlation coefficient −0,467, *p* < 0.05) with the increase in the U1-NA distance (6.29 mm), after the protrusion period (T1-T0, Table 6). The significance has not been found between the remaining parameters (*p* > 0.05).

**Table 6:** Evaluation of the relationship between the upper incisor inclination and pressure measurements

		U1-SN (°)		U1-NA (°)		U1-NA (mm)	
		*r*	*p*	*r*	*p*	*r*	*p*
Upper lip pressure (kPa)	T1-T0	−0.024	0.920	0.038	0.875	0.045	0.850
	T2-T1	−0.132	0.578	−0.181	0.446	−0.400	0.080
Lower lip pressure (kPa)	T1-T0	−0.312	0.181	−0.193	0.414	0.157	0.508
	T2-T1	0.099	0.679	0.157	0.508	0.123	0.604
Vertical lip pressure (kPa)	T1-T0	0.038	0.873	−0.063	0.793	−0.467	0.038*
	T2-T1	0.095	0.690	0.105	0.659	−0.045	0.849

*p < 0.05

## Discussion

Perioral structures play important roles in the development of either a normal occlusion or a malocclusion. Soft tissues like the lips, cheeks, and tongue affect hard tissues and orthodontic treatment results by perioral pressures, muscle forces, and periodontal attachments. Therefore, soft tissue limitations should be assessed more precisely by orthodontists, and they should consider not only the genetics but also the environmental factors.

According to the balance theory defined by Weinstein et al. [16], the teeth are balanced by the tongue from the inside and by the lips and cheeks from the outside. At the same time, even though the magnitude of force is low, it may cause a movement in the teeth when applied for a sufficient amount of time [17–19]. Graber [20] stated that the changes observed in the muscle functions may change the normal morphology or may compound the current malocclusion where he examined the muscle morphologies of the Class I, Class II, and Class III malocclusions. Evaluations regarding the soft tissues are very important for determining the malocclusion etiology and the stability of the orthodontic treatment. Thus, in this study, we aimed for clarifying the relationship between the upper incisor protrusion and perioral pressures.

In the evaluation of the soft tissues, it is possible to investigate thickness or volume measurements as well as the electromyographic or electrodynamic measurements. Applying electrodynamic measurement techniques with strain gauges is a reliable method for evaluating soft tissue forces and pressures [21–31]. Lindeman and Moore [32], comparing three different methods of evaluating the perioral pressure and the force, maintained that the lips cause fluid-like pressure, and thus, they should be evaluated using devices that are sensitive to pressure instead of the force. IOPI is a reliable tool that measures the perioral pressures from the lips, cheeks, and tongue [33–35]. Additionally, there is no need for additional laboratory processes and no risk of contamination because of its single use measurement bulbs.

It was mentioned in previous studies that perioral pressures were affected by the alterations in head posture [6, 36–38]. According to Thüer et al. [6], Ingervall and Thüer [36], and Hellsing and L’Estrange [37], the perioral pressures were increased with the head extension than the natural head position. Therefore, in our study, we paid attention during the pressure records to the maintenance of natural head position.

Consensus about the relationship between the types of malocclusion and perioral pressures has not been achieved yet. Lapatki et al. [10] indicated that the upper incisors were exposed to more resting lip pressure in CII/2 malocclusion compared to Class I occlusion. Oppositely in another study, it has been shown that maximum lip pressure was the lowest in individuals with CII/2 malocclusion [39]. Nevertheless, in both studies, the individuals were older and also the methodology of these studies was different from our study. Thüer and Ingervall [7] found that the ULP on the upper incisors was at the lowest in CII/2 malocclusion and at the highest in Class II division 1 malocclusion. They also mentioned that the LLP did not show significant difference between malocclusions. In a study that included both young and adult individuals, ULP change between Class I and Class II malocclusions did not show significant difference [40]. Additionally, Lambrechts et al. [39] noted that among the types of malocclusion, no significant differences could be found in terms of tongue pressure. In our study, there are no significant pressure differences except LBP between the study and control groups at the beginning of the treatment. This difference disappeared with the decrease of LBP after the protrusion period. However, this decrease has not been thought to result from orthodontic treatment. Though a utility arch has buccal segments, tooth movement apparently occurs at the anterior region. Additionally, since the utility arch is a symmetric appliance, we would wait to see the same effect on the right side. But, a significant change in RBP has not been determined. It was thought that these differences might be due to transitions in dentition, and although attention is shown, bending and inserting the utility arch may be different from one side to the other.

Mitchell and Williamson [41] and Posen [42, 43] stated that the perioral forces increase with age. In the present study, it was seen that ULP increased during the retention period in both groups. The retention period covered a longer phase than the protrusion period. Di Fazio et al. [40] found an increase in the upper lip pressure with age, and he suggested that this increase might be described by the maturation of the orbicularis oris muscle due to growth. Additionally, in another study, it was indicated that the ULP tended to be high in children with a large overjet [7]. These observations might be acceptable for our study.

In the present study, LLP increased and VLP decreased after upper incisor protrusion but these changes did not remain stable in the study group. Some authors [7, 10] stated that the upper incisor position was determined by the increased LLP in CII/2 malocclusion. In one of the studies that investigated the effect of increased overjet in perioral pressures, it was observed that LLP increased [7], while in another study, VLP decreased because tightening of the lips became harder [39]. These findings are compatible with the findings of our study. However, these studies had a cross-sectional study design, and individuals with an increased overjet were included. Meanwhile, in the control group, VLP did not increase significantly after protrusion and then significantly after the protrusion period. Due to these changes, significant difference occurred at the end of protrusion between the two groups. In fact, although not significant, LLP also increased in the control group. All these lip pressure increases observed in the control group might be described as mentioned above in ULP.

The only correlation between the incisor inclination and the lip pressures was found between the increase of the U1-NA distance and the decrease of VLP. This correlation, even though statistically significant, was moderate. However, a significant correlation between the increase of VLP and the decrease of upper incisor inclination was not observed after the retention. Therefore, VLP which did not remain stable after retention was not associated with the mild relapse of upper incisor protrusion. Such as soft tissue morphology changes, other factors that may be effective on the perioral pressures need to be investigated in further studies. Thüer and Ingervall [7] determined that ULP was correlated with the morphology of the lips, while Di Fazio et al. [40] found a significant correlation between ULP and age.

This is the first study to evaluate the perioral pressure changes in terms of lips by IOPI. Furthermore, IOPI can be considered as a diagnostic tool for malocclusions and the progression of myofunctional exercises. Hence, orthodontists can use IOPI for the malocclusions arising from bad oral habits in a practical way. The only limitation of this prospective study was that transitions in mixed dentition might affect the perioral pressure measurements.

It has been known that soft tissues affect skeletal and dentoalveolar hard tissues during growth. Our findings showed that changes that occurred in the anterior teeth had a temporary effect on the soft tissues. Therefore, it should be noted that permanent changes could not be achieved in soft tissue pressures after upper incisor protrusion and relapse resulting from soft tissue pressures should always be kept in mind. In further studies, perioral pressure changes can be examined by including different malocclusions.

## Conclusions

Permanent changes did not occur in perioral pressures with upper incisor protrusion in CII/2 malocclusion. In addition, there is a negative moderate correlation between the protrusion of upper incisors and the vertical lip pressure.

## Abbreviations

CII/2: Class II division 2 malocclusion
IOPI: Iowa Oral Performance Instrument
LBP: Left buccal pressure
LLP: Lower lip pressure
MTP: Maximum tongue pressure
RBP: Right buccal pressure
STP: Swallowing tongue pressure
ULP: Upper lip pressure
VLP: Vertical lip pressure
